# Subject characteristics of medical requests to the Addiction Liaison Psychiatry Unit. 12 years in Hospital del Mar (Barcelona)

**DOI:** 10.1192/j.eurpsy.2023.1380

**Published:** 2023-07-19

**Authors:** J. J. Fuentes Valenzuela, M. García Jimenez, F. Fonseca Casals, M. Torrens Melich

**Affiliations:** 1 Addictions, Institut de Neuropsiquiatria i Adiccions (INAD); 2 Addictions, Institut Hospital del Mar d’Investigacions Mèdiques (IMIM); 3 Departamento de Psiquiatría y Medicina Legal, Universitat Autònoma de Barcelona; 4 Addictions, Institut de Neuropsiquiatria i Addiccions (INAD), Barcelona, Spain

## Abstract

**Introduction:**

Addiction Liaison Pychiatric Units are frequently requested by other medical services due to the high prevalence of medical pathologies in substance use disorders. We intend to know patient’s characteristics in order to improve the approach.

**Objectives:**

To describe patient characteristics of all medical request to the Addiction Liaison Pyshicatric Unit from January 2010 to December 2022.

**Methods:**

Study data will be obtained from all patients that were referred to the Addiction Liaison Psychiatric Unit to assess addictive disorders and withdrawal symptoms related to drugs during 12 years in Hospital del Mar (Barcelona, Spain). Demographics and clinical data (substance use, medical comorbidity and dual diagnosis) were obtained.

**Results:**

The results will be presented as soon as all data is obtained. We will explore COVID-19 pandemic implications.

**Conclusions: Disclosure of Interest:**

None Declared

